# Dietary Diversity and Anthropometric Status of Mother–Child Pairs from *Enset* (False Banana) Staple Areas: A Panel Evidence from Southern Ethiopia

**DOI:** 10.3390/ijerph16122170

**Published:** 2019-06-19

**Authors:** Tafese Bosha, Christine Lambert, Simon Riedel, Aberra Melesse, Hans K. Biesalski

**Affiliations:** 1 Institute of Nutritional Sciences, University of Hohenheim, Garbenstr. 30, 70593 Stuttgart, Germany; christine.lambert@uni-hohenheim.de (C.L.); simon.riedel@uni-hohenheim.de (S.R.); biesal@uni-hohenheim.de (H.K.B.); 2 College of Agriculture, Hawassa University, Hawassa 05, Ethiopia; a_melesse@uni-hohenheim.de

**Keywords:** dietary diversity, anthropometric status, *enset* staple areas, MCP, postharvest dry season, lean wet season, Southern Ethiopia

## Abstract

Background: A sizable cross-sectional studies demonstrated a low dietary diversity in Southern Ethiopia. However, its seasonal trend has not been well studied in areas where nutrient-poor enset (false banana (*Ensete ventricosum*)) foods are major staple. Moreover, there is scarcity of information on seasonal nature of anthropometric status of mother–child pairs (MCP) from the same areas in Southern Ethiopia. Therefore, the present study aimed to investigate the dietary diversity and anthropometric status of MCP in postharvest dry and lean wet seasons and identify factors associated with anthropometric status. Methods: The dietary intake and anthropometric data were collected from 578 households (578 mothers and 578 children) January–June 2017. The study compared data of the two seasons using McNemar’s test for dichotomous, Wilcoxon signed-rank test for non-normally distributed, and paired samples *t*-test for normally distributed continuous data. Logistic regression was conducted to identify risk factors for malnutrition. In addition, Spearman’s Rho test was used to determine correlations between maternal and child variables. Results: Over 94% of the mothers did not fulfil the minimum diet diversity score in both seasons. The meal frequency and pulses/legumes intake significantly declined in lean wet season; however, dark green leaves consumption increased. Meat, poultry, and fish consumption dropped to almost zero in the lean wet season. The dietary diversity and anthropometric status of the MCP were correlated. Weight-for-age (WAZ) and weight-for-height (WHZ) of children significantly declined in the lean wet season. In the same way, maternal mid upper arm circumference (MUAC), body weight, and body mass index (BMI) dropped (*p* < 0.001) in this season. Being pregnant and a lactating mother, poverty, and the ability to make decisions independently predicted maternal undernutrition (low MUAC). On the other hand, maternal undernutrition and education were associated with child underweight. Conclusions: The results demonstrated that the dietary diversity of MCP is low in both postharvest dry and lean wet seasons. This suggests the need for continuous nutrition intervention to improve the dietary diversity. In addition, the anthropometric status of MCP declines in lean wet season. This may provide some clue for policy targeting on improving nutritional status of mothers and children in rural Southern Ethiopia.

## 1. Introduction

Dietary diversity is defined as the number of different foods or food groups consumed over a given reference period [1]. It reflects household access to a variety of foods [2]. The dietary diversity functions as a proxy indicator for nutrient adequacy of diet of individuals [2], particularly that of micronutrients, which is an important dimension of diet quality [3,4]. Its score which is abbreviated as DDS (dietary diversity score), is a count of food groups consumed over certain reference period often in 24 h [2,5].

In order to meet the minimum requirement for a healthy diet, women should obtain more than or equal to five [3], while children above or equal to four food groups over preceding 24 h [6]. However, sizable cross-sectional studies reported a low dietary diversity with limited intake of animal source foods among women [7,8,9] and children [10,11] from Southern Ethiopia. Besides, the major staple in many parts of Southern Ethiopia i.e., *enset* (false banana [*Ensete ventricosum*]) foods are nutrient poor [12,13]. For instance, *bulla,* which is a superior *enset* product, contains 0.65 g protein, 0.22 mg zinc, 7.58 mg iron, and 63.67 mg calcium per 100 g at 7.8% moisture level. On the contrary, 100 g of teff (Eragrostis teff) provides 14.58 g protein, 4.74 mg zinc, 22.37 mg iron, and 146.26 mg calcium on dry matter basis [13]. Similarly, only 1.46 g protein is obtained from 100 g of *kocho (enset* product)-based complementary food; but 8.82 g from that of maize-based counterpart on dry weight basis [12]. Such a diet lacking in animal source foods, fruits, vegetables, and fortified foods causes micronutrients deficiencies [14], which are public health concerns in Ethiopia [15,16,17,18,19,20,21,22,23,24,25,26].

Seasonal food shortage negatively affects the dietary diversity [27]. Also, the literature suggests that it is helpful to measure the DDS in different seasons as it is a proxy indicator of micronutrient intake [28]. However, a rare study on seasonality of household diet in Ethiopia used household, consumption, and expenditure survey (HCES) data which was not originally designed for nutrition analysis [29]. Moreover, there is a scarcity of information on the seasonal trends of dietary diversity of women and children from *enset* food-consuming communities in Southern Ethiopia for right intervention.

According to UNICEF, inadequate food intake is the number one and immediate cause of child undernutrition [30]. In general, child and maternal undernutrition has been a serious issue in Sub-Saharan Africa, including Ethiopia. This can be exemplified by the fact that 27% of Ethiopian reproductive age women have body mass index (BMI) < 18.5 [31]. Likewise, nearly 25% of the under-five children are underweight and 10% are wasted (thin for height) [32]. Undernutrition poses a substantial social and economic costs. For example, maternal and child undernutrition is an underlying cause of 3.5 million deaths and 35% of the disease burden in under-five children globally [33]. When it comes to Ethiopia, the child undernutrition alone causes 28% of all child deaths and loss of estimated 16.5% of gross domestic product (GDP) [34]. Among others, child underweight (low weight for age) has been a leading risk factor for most disease burdens in the country [35].

Limited literatures from other settings showed the increase in prevalence of undernutrition in the annual lean period when the previous year’s harvest stocks deplete [27,36]. However, such studies are not only very few in the country, but also ended up with less consistent conclusion. Additionally, trends of anthropometric status of mother–child pairs (MCP) specifically from *enset* foods-consuming communities needed investigation during postharvest dry and lean wet season. Therefore, this panel study aimed to investigate the seasonal trends of dietary diversity and anthropometric status of MCP from *enset* staple areas of Southern Ethiopia and identify factors associated with anthropometric status.

## 2. Materials and Methods 

### 2.1. Study Area

This study was conducted in Shebedino and Hula Districts from Sidama Zone in Southern Nations Nationalities and Peoples Region (SNNPR). The SNNPR contains 56 ethnic groups reflecting extreme diversity with unique social and cultural identities [37]. The region is known for *enset* production and *enset* food products are amongst its typical traditional foods and staples. Sidama is one among the 15 zones in this region. According to 2017 population projection, Sidama Zone has a total population of nearly 3.1 million [38]. Coffee, *enset,* and maize are amongst the major crops in this zone.

### 2.2. Study Design

This is a community-based panel study. Dietary diversity and anthropometric status of MCPs were assessed. Data collection was conducted in postharvest dry season (January to first week of February 2017) and lean wet season (June 2017).

### 2.3. Study Participants

Reproductive age mothers and 24–59-month-old children were eligible for this study. In order to reduce the loss to follow-up, temporary-resident mothers and children were excluded from this study. Furthermore, the mothers and children who were severely sick during the survey periods were excluded as sickness alters the dietary intake pattern.

### 2.4. Sample Size Determination and Sampling Procedure 

For this study, a sample size of 492 was calculated using a single population proportion formula. In the calculation, 0.05 degree of precision, 1.5 effect design, and 0.27 expected prevalence of BMI < 18.5 kg/m^2^ were considered [31]. Finally, 10% of basic sample size was added to compensate for the loss to follow-up. To obtain a sufficient sample size for additional analysis, a total of 625 MCP were enrolled in postharvest dry season. 

A two-stage sampling technique was employed. Firstly, two *enset* growing districts (Shebedino and Hula) were selected from Sidama Zone. Secondly, five kebeles (kebele is the smallest administrative unit of district) were randomly selected using probability proportional to size (PPS). Then, the sample size was distributed to each kebele using PPS. Accordingly, 239 MCP was sampled from Hula District (Worare 155 MCP and Chirone 84 MCP) and 386 MCP from Shebedino District (Fura 126 MCP, Howolso 124 MCP, and Dila Gumbe 136 MCP). The MCP was randomly selected based on the sampling frame prepared from house-to-house listing of households with 24–59-month-old children. From the initially registered total, 578 MCP completed the study. However, 47 left the study due to delivery, migration, death, and severe illness during the lean wet season data collection.

### 2.5. Data Collection

#### 2.5.1. Anthropometry

For body weight measurement, portable digital scale (Seca 770, Hanover, Germany) was used. Body weight was measured with light wear to minimize weight effect of over coats. Mothers’ height was measured with dissembling plastic height measuring board with a sliding head bar. A Shorr sliding length board was used to measure standing height of children. The study participants were requested to take off their shoes to minimize its effect on height measurement. Mid upper arm circumference (MUAC) of mothers was measured using MUAC tape. Height and MUAC were measured to the nearest 0.1 cm and weight to the nearest 0.01 kg. Measurements were taken in duplicate and averaged. When the variation was above maximum tolerable difference (0.5 kg in weight, 1.0 cm in height, and 0.5 cm in MUAC), a third measurement took place. In order to assess the seasonal trends of anthropometric status, z-scores of weight-for-age (WAZ) and weight-for-height (WHZ) were computed for children. Next, BMI of the mothers was calculated as weight in kilograms divided by the square of their height in meters [39]. To identify factors associated with anthropometric status of the MCP, the children with WAZ < −2 SD were categorized as underweight while the ones with WAZ ≥ −2 SD as normal [39]. As the BMI was not appropriate for maternal categorization due to the weight gain of pregnant women, MUAC was used. Accordingly, the mothers were categorized as undernourished when MUAC < 22 cm for non-pregnant/non-lactating, and normal when MUAC ≥ 22 cm [40]. Pregnant and lactating mothers with MUAC < 23 cm were classified undernourished, and normal when MUAC ≥ 23 cm [40,41].

#### 2.5.2. Wealth Index

Household’s wealth status was assessed collecting data on variables related to ownership of valuable assets, type of living house, and possession of improved water and sanitation facilities. For principal component analysis (PCA), 18 variables (types of roof and floor, number of sleeping rooms, availability of window, availability of separate kitchen, owning cow/ox, sheep, goat, transport animals (horse/mule and/or donkey), source of cooking fuel, cleaning drinking water, saving account, radio, mobile, chair, bed, land size, and electricity) were used in this study. The categorical variables were recoded into dichotomous indicators (0, bad; 1, improved). Then, PCA was analyzed to produce common factor scores. The first factor, which explained 66.30% of the variation, was taken to characterize the households’ wealth status. Finally, the households were ranked as five wealth quintiles (highest, second, middle, fourth, and lowest) [32].

#### 2.5.3. Household Food Security Status 

The household food insecurity access scale (HFIAS) status indicator questionnaire was used to determine the degree of food insecurity (access) in the households. The HFIAS questionnaire contained nine main questions. Each main question was followed by a sub-question on frequency of occurrence of a condition in the past four weeks (1, rarely; 2, sometimes; 3, often). Based on the HFIAS indicators, the households were categorized into four groups (food secure, mild, moderately, and severely food insecure) [42].

#### 2.5.4. Dietary Intake

In order to capture the usual intake, dietary consumption data were collected using 24 h dietary recalls in days other than fasting and holidays. In addition, mothers and children who were severely sick at the time of the survey were excluded to avoid effect of illness on the usual dietary pattern.

Food models, food charts, and local units were used to facilitate mothers’ recall. Mothers’ DDS was constructed based on 10 food groups (grains, white roots, and tubers; pulses (beans, peas, and lentils); nuts and seeds; dairy; meat, poultry and fish; eggs; dark green leafy vegetables; other vitamin A-rich fruits and vegetables; other vegetables; and other fruits) [3]. Children’s DDS was created based on 7 food groups (grains, roots, and tubers; legumes and nuts; dairy products (milk, yogurt, cheese); flesh foods (meat, fish, poultry, and liver/organ meats); eggs; vitamin-A rich fruits and vegetables; and other fruits and vegetables) [6]. Food groups were assigned to 1 if any food item within the group consumed, otherwise 0, if not eaten in the last 24 h. Negligible food items with intake less than 15 g/day were not considered in the current study. A cutoff point of ≥5 was used to determine minimum dietary diversity score (MDDS-W) for mothers [3]. On the other hand, a cutoff point of ≥4 was used to compute minimum dietary diversity score of children (MDDS-C) [6].

### 2.6. Data Quality Control 

A structured and semi-structured questionnaire was prepared first in English, then translated to Amharic. Enumerators with educational background of health, nutrition, and agriculture who have experience of data collection were recruited. Data collectors were trained by the principal investigator for one week. Similar enumerators collected the lean wet season data after a refreshing training. The data collection instruments were pre-tested on 5% of the sample size in other kebele for the necessary adjustment. Anthropometric measurements were taken by the nutritionists from Hawassa University. Data collection was supervised by the principal investigator. 

### 2.7. Data Analysis

Before data entry, data templates were prepared, and variables were coded. Following, data were entered into SPSS version 20 (IBM Corporation, Armonk, NY, USA) for analysis. Children’s WHZ and WAZ were analyzed using WHO Anthro version 3.2.2 (World Health Organization, Geneva, Switzerland). Data normality was checked with Kolmogorov–Smirnov test. To compare the postharvest dry and lean wet seasons, Wilcoxon signed-rank test was used for non-normally distributed data (DDS, animal source food consumption (ASF), meal frequency, *enset* food intake, MUAC, and BMI). Additionally, a paired samples *t*-test and McNemar’s test were employed to compare normally distributed continuous (WAZ and WHZ) and dichotomous data (food group consumption), respectively. Logistic regression was conducted to identify association between the outcomes (maternal undernutrition and child underweight) and independent (socioeconomic and dietary intake related) variables. Univariate logistic regression was conducted to identify independent variables, which were candidates for multivariate multiple logistic regression. The independent variables with *p*-value < 0.25 were entered into the final logistic regression model to adjust for confounders. Multi-collinearity of the variables was checked with the variance inflation factor and standard error with respective cutoff points of <10 and <2. The Hosmer and Lemeshow test was used to check for goodness of fit for the final model. Crude and adjusted odds ratios were reported with 95% confidence interval to show the strength of association between the outcomes and predictors. The cutoff point for statistical significance test was a p-value of 0.05.

### 2.8. Ethical Consideration

Ethical clearance was received from Institutional Review Board of Hawassa University, Ethiopia; and Ethik-Kommission, Landesäztekammer Baden-Württemberg, Germany (F-2016-127). Supplementary permission was obtained from health administrative offices of the study districts. Written informed consent was received from mothers. Information was kept confidential by providing pseudonymous codes. 

## 3. Results

### 3.1. Sociodemographic Characteristics of Study Participant MCP 

The study participant mothers were 20–40 years old. Majority of the mothers were from Sidama ethnicity. From the study participants, 98% were married during this survey. The mean family size was 5.2. From the children participated in this study, 34% were 24–35 months old, nearly 49% were 36–47 months, and almost 17% were 48–59 months old. Educational attainment of the mothers was low with 41% illiteracy rate. Nearly 88% of the mothers were housewives. Almost all produced *enset* in the last 12 months before the study. The results showed that 74% of the households were moderately food insecure, while 20% were in the poorest wealth quantile (Table 1).

### 3.2. Comparison of Food Consumption of MCP between Postharvest Dry and Lean Wet Seasons

Table 2 demonstrates variations in food group consumption in MCP in postharvest dry and lean wet seasons. The consumption of pulses/legumes significantly reduced; but that of dark green leafy vegetables increased (*p* < 0.001) in lean wet season. However, eggs, nuts, and seeds were not consumed by almost all mothers in the two seasons. Very few children consumed eggs in the lean wet season. Besides, meat, fish, and poultry intake of MCP was very low in postharvest dry and almost absent in lean wet season. Similar to mothers, intake of pulses/legumes was significantly decreased in lean wet season in children; but that of vitamin A-rich vegetables increased (*p* < 0.001).

### 3.3. Comparison of Dietary Diversity and Anthropometric Status of MCP in Postharvest Dry and Lean Wet Season

This study found that the majority of the mothers (>94%) did not fulfil the MDD-W in both postharvest dry and lean wet seasons. Simultaneously, nearly 59% and 65% of the children did not fulfill the MDDS-C, respectively, in postharvest dry and lean wet season. More than half of the MCP obtained ≤3 DDS in both seasons. The maternal ASF intake was very low (~21%); but over 81% consumed *enset* food products across seasons. About 40% of the mothers consumed less or equal to three meals per day in postharvest dry season; but this increased to 46% in lean wet season (Table 3). 

The seasonal comparison of dietary diversity and anthropometric status of MCP is presented in Table 4. The mean DDS of MCP remained very low without a significant change in postharvest dry and lean wet seasons. The meal frequency of the MCP was significantly decreased (*p* < 0.001) in lean wet season. The intake of ASF decreased in children during lean wet season but did not show a difference in mothers. Contrarily, percent of mothers that consumed *enset* food products significantly decreased in lean wet season; but such change was not seen in children.

Maternal MUAC dropped significantly (*p* < 0.001) during the lean wet season. Similarly, the maternal mean body weight and BMI (except for pregnant women) and children’s anthropometric status declined in this season (Table 4). 

Table 5 displays correlations between dietary diversity and anthropometric status of MCP. The study identified a significant correlation in DDS (r = 0.32, *p* < 0.01) and meal frequency (r = 0.27, *p* < 0.01) of MCP. The findings demonstrate a high similarity in trends of MCP dietary diversity. Also, the child underweight and maternal undernutrition (low MUAC) showed a significant correlation (r = 0.11, *p* < 0.01) showing a higher probability for a child to be underweight when his/her mother becomes undernourished. 

### 3.4. Factors Associated with Anthropometric Status of MCP 

Compared with the mothers neither pregnant nor lactating, pregnant women had extremely high odds for undernutrition (low MUAC) (AOR = 10.92 (CI: 1.88, 63.26)). Lactation and poverty independently predicted the maternal undernutrition. Mothers from male-headed households were 1.92 times more exposed for undernutrition compared with those involved in decision making over the household’s resources. Child underweight was predicted by maternal undernutrition and educational level. However, household food insecurity, child age, family size, distance to water source, DDS, meal frequency, intakes of *enset* food products, and animal source food did not show significant association with anthropometric status of MCP (Table 6).

## 4. Discussion

This study assessed whether or not the dietary diversity and anthropometric status of mother–child pairs from *enset* staple areas are different in postharvest dry and lean wet seasons. The study also investigated factors associated with anthropometric status. Accordingly, the findings are discussed in the following section.

### 4.1. Dietary Diversity of Mother–Child Pairs 

A cross-sectional study from Angecha District of Southern Ethiopia reported that 52% of the lactating mothers have low diet diversity [7]. Further study on pregnant mothers from Lemo District in the same region found a low diet diversity prevalence of 47% [9]. The current finding, i.e., 64%, is the highest compared with the past reports [7,9]. There are several reasons for this discrepancy. The variation in agro-ecology together with the differences in dietary pattern and data collection interval could be responsible for the discrepancies. Furthermore, intake of carbohydrate source foods was found to highly indicate monotonous nature of the diet (Table 2). Such a low dietary diversity implies a poor diet quality and inadequate intake of micronutrients [3,4]. Therefore, interventions to increase fruits and vegetables intake and nutrition education could help to improve the dietary diversity.

Nearly 41% of the study participant children met minimum diet diversity score; but this percentage dropped to 35% in lean wet season. Nonetheless, it is still higher compared with the research report from Afar on 6–59-month-old children (~31%) [43], and Wolaita Sodo town on 6–23-month-old children (~27%) [10]. The present finding also contradicts with the report on 6-23-month-old children sampled from Eastern and Northern parts of Ethiopia [44]. These discrepancies can be explained by the variations in agro-ecology, fruits and vegetables production, dietary pattern, age of children, and data collection period. 

The decline in meal frequency during lean wet season (Table 4) is attributable to a low availability of food from subsistent farming, increased food purchase, and higher food price in this season [45]. Similar reasons could hold true for the fall in consumption of pulses/legumes in lean season (Table 2). The reduction in pulse/legume intake could contribute to a decrease in protein and zinc intake as they are better than *enset* foods in terms of protein and zinc contents. For example, a 100 g of faba bean contains 27 g protein and 4.1 mg zinc on dry weight basis [46]. Disparately, bulla, which is a superior *enset* product, contains only 0.65 g protein and 0.22 mg zinc per 100 g at 7.8% moisture level [13]. 

The fact that majority of the mothers (>94%) could not meet minimum diet diversity score may be due to a low intake of animal source food (ASF) (Table 2) as they showed a positive correlation (Table 5).

The national food consumption survey reported little contribution of meat (0.4%) and eggs (0.2%) to the women’s diet in Southern Ethiopia. Alike, their share was reported to be low in children’s diet (meat 1.0% and eggs 1.1%) [47], which support the current findings. The possible explanations for the low intake of ASF could be that eggs are sold to cover children’s schooling and other expenses. Fish is not consumed by the study participants because of its low availability in markets due to lack of cold storage, unavailability of dried fish, and long transportation. Further, most of the rural households are unable to afford the high price of meat. Such a low intake of ASF could result in reduced protein and micronutrient intake. Therefore, interventions improving income of the households and small animal farming could improve access to ASF.

Little or no consumption of nuts and seeds like flaxseed, sunflower, safflowers, and sesame recorded in this study may be due to their low availability as such food items are produced rarely in the study area. When they are low in diet, the intake of protein and micronutrients could be reduced. 

A research study from Ghana reported a significant increase in vitamin A-rich vegetable consumption among school age children in rainy season [28]. This report is in agreement with the current results. The increase in vitamin A rich vegetable intake is related to a high availability of green leaves like Ethiopian kale (*Brassica carinata*) in lean wet season. Such an increased vegetable consumption could improve the dietary intake of vitamin A and reduce its deficiency problems. 

Overall, diets of the mother–child pairs were dominated by carbohydrate source foods in both postharvest dry and lean wet seasons. Moreover, the meal frequency, and percentage of the population that meet requirement of minimum diet diversity score decrease in lean wet season. The implication of these findings is that the study population has an increased chance of exposure for malnutrition in lean wet season, so that appropriate intervention is necessary.

### 4.2. Anthropometric Status of Mother–Child Pairs

Egata and colleagues found that wet season predicts acute undernutrition in 6–36-month-old children from Eastern Ethiopia [48]. Another study on 6–23-month-old children from Ethiopia reported a decrease in wasting; but an increase in underweight in pre-harvest (lean season) which sound conflicting [44]. Maybe this is because the second study was completed on a lower sample size. Despite that, the current decline in weight-for-height z-score is supported by the first [48], while drop in weight-for-age is backed by the second study [44].

Similar to the children, the maternal anthropometric status was declined in lean wet season (Table 4). The current finding is supported by the previous report on lactating mothers from Ethiopia [49] and another one from Burkina Faso [27]. This might be due to a decrease in the calorie intake as a consequence of reduced food availability, increased food purchase, and high price in this season [29,45]. The observed high odds of maternal undernutrition (low MUAC) in pregnant and lactating women (Table 6) could be possibly explained by a low intake of energy recorded in pregnant mothers from the same study zone [50,51].

The fact that mothers from male-headed households have a higher risk for low MUAC may be associated with their ability to make decision over the household’s resource utilization and sharing. Therefore, women’s capacity building and enabling them to make decisions could help in reducing this problem.

In the present study, poverty predicted maternal low MUAC, which is supported by the earlier report from Lemo District in Ethiopia [9]. This is possibly because diet of the mothers from poor households is mostly monotonous, which is dominated by starchy staple with a low meal frequency.

Maternal education protects child undernutrition [52,53] as also seen in the present study. This is because education increases maternal awareness so that their children can obtain better feeding and caring services.

A cross-sectional study from Hula District in Ethiopia reported association between child weight-for-height z-score and maternal BMI [54]. Also, a low maternal BMI predicted child wasting in Ghana [55]. These prior reports support the present results. Maybe this is because dietary diversity, meal frequency, and anthropometric status of the mothers and their children are correlated (Table 5).

In summary, first, the dietary diversity did not show association with anthropometric status of the mother–child pairs. A possible reason for this is that the dietary diversity is proxy mostly for micronutrient quality of the diet [3,4]. Second, some possible exogenous shocks like political instability and natural disaster, which could impact average results between the two seasons investigated, may not substantially affect our conclusion. This is because most of the acute hunger and undernutrition occur in an annual lean season when the previous harvest stocks are exhausted [36].

This study has several strengths and few weaknesses. First, this is a panel study and compared data from mothers and children from similar households in postharvest dry and lean wet season. Second, the study targeted on areas where a nutrient-poor *enset* is a staple food. Third, this study focused on mothers, especially lactating and pregnant and under-five children who are at risk for malnutrition in the society. Fourth, it determined the variations in dietary diversity and anthropometric status of mother–child pairs in postharvest dry and lean wet season. Last, the large sample size makes the findings more generalizable and conclusive. However, the results of this study show the outcomes of qualitative undernutrition. More specifically, the low mid-upper-arm circumference, low body mass index, and underweight reflect inadequate energy intake from diet. The dietary diversity is a proxy for micronutrient quality of the diet. Therefore, we recommend further studies on biomarkers to determine the trends of nutrient deficiencies by season.

## 5. Conclusions

This study demonstrated that the dietary diversity of mother–child pairs is low in both postharvest dry and lean wet season in *enset* staple areas of Southern Ethiopia. Moreover, intake of animal source foods, which are good sources of protein and micronutrients, is generally low in both seasons and, especially, meat and eggs are almost absent in the lean wet season. Therefore, a continuous nutrition intervention targeting on improving dietary diversity is vital for the community. It has been also found that anthropometric status of mother–child pairs declines in lean wet season. Besides, the maternal undernutrition (low MUAC) and education independently predicted child underweight. Whereas, poverty, physiological status of mothers, and ability of decision making on household’s resource were associated with the maternal undernutrition. These results may provide some hints for policy targeting on improving maternal and child nutritional status in rural Southern Ethiopia. The findings suggest that pregnant and lactating women need priority for interventions.

## Figures and Tables

**Table 1:** Sociodemographic characteristics of the study’s participating mother–child pairs (MCP) (January–June 2017).

Characteristics (n = 578)	Category	Number	Percent
Education status	Illiterate	239	41.3
	Grade 1–4	140	24.2
	Grade 5–8	150	26.0
	≥grade nine	49	8.5
Occupation	Housewife	507	87.7
	Petty trade	56	9.7
	Other	15	2.6
*Enset* production in the last 12 months	Produced	570	98.6
	Did not produce	8	1.4
Household food security status	Food secured	53	9.2
	Mildly food insecure	41	7.1
	Moderately food insecure	429	74.2
	Severely food insecure	55	9.5
Wealth quantiles	Highest	116	20.1
	Second	115	19.9
	Middle	116	20.1
	Fourth	117	20.2
	Lowest	114	19.7
Ethnicity	Sidama	561	97.1
	Other	17	2.9
Religion	Protestant	529	91.5
	Muslim	34	5.9
	Orthodox	8	1.4
	Catholic	7	1.2
Marital status	Co-habiting in marriage	564	97.6
	Other	14	2.4
Family size	3–4 members	239	41.3
	5–6 members	216	37.4
	≥7 members	123	21.3
Age of children, months	24–35	199	34.4
	36–47	282	48.8
	48–59	97	16.8
Sex	Boys	287	49.7
	Girls	291	50.3

**Table 2:** Comparison of food group consumption in MCP between postharvest dry and lean wet seasons (N = 578), January–June 2017.

	Food Groups	Dry Season No. (%)	Wet Season No. (%)	*p*-Value
Mothers	Grains, roots and tubers	578 (100.0)	578 (100.0)	NA
	Pulses/legumes	359 (62.1)	201 (34.8)	<0.001
	Nuts and seeds	1 (0.2)	0 (0.0)	1.000 *
	Dairy	121 (20.9)	124 (21.5)	0.884
	Meat, poultry and fish	9 (1.6)	1 (0.2)	0.021
	Eggs	0 (0.0)	0 (0.0)	NA
	Dark green leafy vegetables	310 (53.6)	464 (80.3)	<0.001
	Other vitamin A-rich, fruits and vegetables	0 (0.0)	1 (0.2)	1.000 *
	Other vegetables	444 (76.8)	479 (82.9)	0.013
	Other fruits	25 (4.3)	29 (5.0)	0.665
Children	Grains, roots and tubers	578 (100.0)	578 (100.0)	NA
	Pulses/legumes/nuts	315 54.5)	193 (33.4)	<0.001
	Milk and milk products	199 (34.4)	172 (29.8)	0.099
	Meat, poultry and fish	11 (1.9)	0 (0.0)	0.001
	Eggs	25 (4.3)	5 (0.9)	<0.001
	Vitamin A-rich plant foods	286 (49.5)	436 (75.4)	<0.001
	Other fruits and vegetables	466 (80.6)	476 (82.4)	0.493

McNemar’s test was used for analysis; N: number; NA: not applicable for the data; paired differences is significant at *p*-value 0.05. * = almost completely similar.

**Table 3:** Descriptive statics of dietary diversity of MCP in postharvest dry and lean wet seasons (N = 578), January–June 2017.

Variables	Category	Mothers		Children	
		Postharvest Dry Season N (%)	Lean Wet Season N (%)	Postharvest Dry Season N (%)	Lean Wet Season N (%)
DDS	≤3	370 (64.0)	373 (64.5)	339 (58.7)	377 (65.2)
	4–5	208 (36.0)	203 (35.1)	239 (41.3)	200 (34.6)
	≥6	0 (0.0)	2 (0.3)	0 (0.0)	1 (0.2)
MDDS-W/MDDS-C	Fulfilled	27 (4.7)	34 (5.9)	239 (41.3)	201 (34.8)
	Unfulfilled	551 (95.3)	544 (94.1)	339 (58.7)	377 (65.2)
Number of meals	≤3	230 (39.8)	264 (45.7)	42 (7.3)	150 (26.0)
	4–5	346 (59.9)	314 (54.3)	531 (91.9)	425 (73.5)
	≥6	6 (0.3)	0 (0.0)	5 (0.9)	3 (0.5)
*Enset* food	Consumed	522 (90.3)	488 (84.4)	468 (81.0)	483 (83.6)
	Did not consume	56 (9.7)	90 (15.6)	110 (19.0)	95 (16.4)
ASF	Consumed	130 (22.5)	125 (21.6)	222 (38.4)	173 (29.9)
	Did not consume	448 (77.5)	453 (78.4)	356 (61.6)	405 (70.1)

N: number; DDS: diet diversity score; MDDS-W: minimum diet diversity score for women; MDDS-C: minimum diet diversity score for children; ASF: animal source food.

**Table 4:** Comparison of dietary diversity and anthropometric status of MCP between postharvest dry and lean wet seasons (N = 578), January–June 2017.

Variables	Mothers			Children		
	Postharvest Dry Season	Lean Wet Season	Sig.	Postharvest Dry Season	Lean Wet Season	*p*-Value
DDS, mean (SD) ^a^	3.2 (0.7)	3.26 (0.8)	0.195	3.3 (0.9)	3.2 (0.9)	0.313
Number of meals, mean (SD) ^a^	3.8 (0.8)	3.61 (0.7)	<0.001	4.6 (0.6)	4.1 (0.8)	<0.001
*Enset* food intake, N (%) ^c^	522 (90.3)	488 (84.4)	0.001	468 (81.0)	483 (83.6)	0.235
ASF intake, N (%) ^c^	130 (22.5)	125 (21.6)	0.773	222 (38.4)	173 (30.0)	0.003
Children’s WHZ, mean (SD) ^b^	-	-	-	−0.15 (0.9)	−0.26 (1.0)	<0.001
Children’s WAZ, mean (SD) ^b^	-	-	-	−0.87 (1.1)	−1.02 (1.1)	<0.001
Maternal MUAC, mean (SD) ^a^	24.4 (2.3)	24.0 (2.3)	<0.001	-	-	-
Maternal body weight (kg), mean (SD) *^,a^	49.6 (6.3)	49.3 (6.5)	0.003	-	-	-
Maternal BMI, mean (SD) *^,a^	20.5 (2.1)	20.4 (2.6)	0.002	-	-	-

^a^ = Wilcoxon signed-rank test was used for analysis; ^b^ = Paired samples t-test was used for analysis; ^c^ = McNemar’s test was used for data analysis; N: number; * = pregnant mothers were excluded from analysis (n = 460); DDS: dietary diversity score; ASF: animal source food; WHZ: weight-for-height z-score; WAZ: weight-for-age z-score; BMI: body mass index; paired difference is significant at *p*-value 0.05.

**Table 5:** Correlation of dietary diversity and anthropometric status of MCP (N = 578), January–June 2017.

	Child Underweight	Child DDS	Child ASF Intake	Child *Enset* Food Intake	Child Meal Frequency	Maternal Undernutrition (Low MUAC)	Maternal DDS	Maternal ASF Intake	Maternal *Enset* Food Intake	Maternal Meal Frequency
Child underweight	1									
Child DDS	0.02	1								
Child ASF intake	0.06	0.39 **	1							
Child *enset* food intake	0.04	0.03	0.05	1						
Child meal frequency	0.07	0.03	−0.04	-0.05	1					
Maternal undernutrition	0.11 **	0.06	0.02	0.01	−0.01	1				
Maternal DDS	0.05	0.32 **	0.14 **	−0.09 *	0.04	−0.00	1			
Maternal ASF intake	0.04	0.16 **	0.52 **	0.07	-0.03	0.05	0.33 **	1		
Maternal *enset* food intake	0.08	0.05	0.09 *	0.41 **	-0.02	0.00	0.02	0.08	1	
Maternal meal frequency	0.07	0.00	0.10^*^	−0.05	0.27 **	0.02	0.09 *	0.07	0.04	1

Spearman’s Rho correlation test was used for analysis; N: number; ASF: animal source food; DDS: dietary diversity score; * correlation is significant at *p*-value 0.05 (2-tailed); ** correlation is significant at *p*-value 0.01 (2-tailed).

**Table 6:** Association of socioeconomic and dietary intake related variables with anthropometric status of MCP (N = 578).

Independent Variables	Outcome Variables					
	Maternal Undernutrition (Low MUAC)			Child Underweight		
	COR (95% CI)	AOR (95% CI)	Sig.	COR (95% CI)	AOR (95% CI)	*p*-Value
Age of children						
24–35 months	1	1		1	1	
36–47 months	0.62 (0.40, 0.97) *	0.74 (0.46, 1.76)	0.199	0.67 (0.42, 1.08)	0.68 (0.41, 1.13)	0.138
48–59 months	0.83 (0.47, 1.47)	1.22 (0.64, 2.32)	0.550	0.49 (0.24, 1.01) *	0.48 (0.22, 1.06)	0.069
Maternal anthropometric status	NA					
Normal	-	-	-	1	1	
Undernourished (low MUAC)	-	-	-	1.92 (1.18, 3.13) *	1.79 (1.07, 3.00)	0.028
Maternal physiological status						
Neither pregnant nor lactating	1	1		1	1	
Pregnant	9.62 (1.76, 52.59) *	10.92 (1.88, 63.26)	0.008	0.00 (0.00, -)	0.00 (0.00, -)	0.999
Lactating	3.23 (1.75, 5.94) *	3.33 (1.72, 6.43)	<0.001	1.83 (1.01, 3.29) *	1.31 (0.69, 2.50)	0.411
Maternal education status						
Illiterate	1	NE		1	1	
1–4th grade	0.86 (0.51, 1.43)	-	-	0.53 (0.31, 0.95) *	0.62 (0.34, 1.12)	0.112
5–8th grade	0.93 (0.57, 1.52)	-	-	0.38 (0.21, 0.70) *	0.44 (0.23, 0.86)	0.016
≥9th grade	0.99 (0.48, 2.07)	-	-	0.48 (0.19, 1.18)	0.61 (0.23, 1.64)	0.328
Decision maker on household’s resource						
Both mother and father	1	1		1	NE	
Mother	2.41 (0.87, 6.67)	2.47 (0.84, 7.27)	0.100	0.90 (0.25, 3.22)	-	-
Father	1.97 (1.27, 3.03) *	1.92 (1.21, 3.05)	0.006	0.99 (0.63, 1.56)	-	-
Family size						
3–4 members	1	1		1	1	
5–6 members	0.73 (0.47, 1.17)	0.74 (0.45, 1.20)	0.225	1.63 (0.98, 2.72)	1.51 (0.86, 2.64)	0.150
≥7 members	1.01 (0.60, 1.68)	1.06 (0.60, 1.84)	0.849	1.60 (0.86, 2.90)	1.30 (0.66, 2.54)	0.451
One way distance to water source						
<30 min	1	1		1	1	
≥30 min	1.29 (0.86, 1.95)	1.09 (0.71, 1.69)	0.686	1.39 (0.88, 2.19)	1.21 (0.75, 1.96)	0.431
Household food security status						
Food secured	1	1		1	1	
Mild food insecurity	1.35 (0.43, 4.22)	1.24 (0.37, 4.14)	0.728	3.43 (0.83, 14.21)	2.80 (0.64, 12.19)	0.170
Moderate food insecurity	1.84 (0.81, 4.22)	1.23 (0.49, 3.06)	0.656	3.53 (1.07, 11.62) *	2.51 (0.730, 8.65)	0.144
Severe food insecurity	2.94 (1.04, 7.83) *	2.07 (0.71, 6.08)	0.185	3.26 (0.83, 12.79)	2.21 (0.53, 9.26)	0.279
Wealth index						
Highest	1	1		1	1	
Second	2.55 (1.25, 5.22) *	2.64 (1.23, 5.66)	0.013	1.77 (0.84, 3.73)	1.56 (0.70, 3.45)	0.278
Middle	2.64 (1.29, 5.39) *	2.66 (1.24, 5.71)	0.012	1.36 (0.63, 2.95)	1.19 (0.52, 2.76)	0.676
Fourth	2.38 (1.16, 4.88) *	2.45 (1.04, 4.88)	0.041	2.64 (1.10, 4.67) *	1.65 (0.75, 3.61)	0.214
Lowest	2.58 (1.26, 5.29) *	2.19 (1.00, 4.79)	0.050	1.39 (0.64, 3.01)	1.01 (0.43, 2.36)	0.979
*Enset* foods						
Did not consume	1	NE		1	NE	
Consumed	1.01 (0.52, 1.98)	-	-	1.29 (0.71, 2.33)	-	-
Animal source food						
Consumed	1	1		1	1	
Did not consume	0.76 (0.48, 1.20)	0.87 (0.54, 1.42)	0.584	0.73 (0.47, 1.15)	0.70 (0.44, 1.12)	0.139
DDS						
4–6	1	NE		1	NE	
<3	0.96 (0.63, 1.44)	-	-	1.05 (0.67, 1.64)	-	-
Meal frequency		NE			NE	
4–6	1			1		
<3	0.89 (0.59, 1.34)	-	-	0.68 (0.26, 1.78)	-	-

COR: crude odds ratio; AOR: adjusted odds ratio; CI: confidence interval; DDS: dietary diversity score; N: number; Hosmer and Lemeshow test *p*-value was 0.320 for final model of mothers and 0.760 for children’s final model; NE: not entered in final model as *p*-value >0.250 in univariate analysis; * = association between the outcomes and predictors is significant at *p*-value 0.05.
